# γ-Tubulin–γ-Tubulin Interactions as the Basis for the Formation of a Meshwork

**DOI:** 10.3390/ijms19103245

**Published:** 2018-10-19

**Authors:** Catalina Ana Rosselló, Lisa Lindström, Greta Eklund, Matthieu Corvaisier, Maria Alvarado Kristensson

**Affiliations:** Molecular Pathology, Department of Translational Medicine, Lund University, Skåne University Hospital, 20502 Malmö, Sweden; ca.rossello@uib.es (C.A.R.); lindstromlisa88@gmail.com (L.L.); greta@rwi.se (G.E.); matthieu.corvaisier@med.lu.se (M.C.)

**Keywords:** γ-tubulin, meshwork, γ-tubules, γ-strings, γ-TuRC, γ-TuSC

## Abstract

In cytoplasm, protein γ-tubulin joins with various γ-tubulin complex proteins (GCPs) to form a heterotetramer γ-tubulin small complex (γ-TuSC) that can grow into a ring-shaped structure called the γ-tubulin ring complex (γ-TuRC). Both γ-TuSC and γ-TuRC are required for microtubule nucleation. Recent knowledge on γ-tubulin with regard to its cellular functions beyond participation in its creation of microtubules suggests that this protein forms a cellular meshwork. The present review summarizes the recognized functions of γ-tubulin and aims to unite the current views on this protein.

## 1. Introduction

Microtubules are highly enriched in a family of GTPases called the tubulins. In humans, there are five known tubulin isoforms: α-tubulin, β-tubulin, γ-tubulin, δ-tubulin, and ε-tubulin [1]. Among the members of the tubulin GTPase superfamily, only the α-, β-, and γ-tubulins are ubiquitous, implying that these proteins perform essential cellular functions that might be conserved throughout the eukaryotes. The first tubulin genes were discovered in the 1970s [2], and since then several genes coding for α- and β-tubulin have been described. In humans, α- and β-tubulin are encoded by 19 genes (10 and 9 genes, respectively) (http://genome.ucsc.edu/) [3]. Subsequent research identified γ-tubulin as a regulator of microtubule assembly in the fungus *Aspergillus nidulans* [4]. In eukaryotes, the number of genes encoding for α- and for β-tubulin varies considerably, whereas the number encoding γ-tubulin ranges from 1 to 3, with two genes found in mammals and up to three genes in flowering plants [3]. The disparity in the number of genes encoding the various members of the tubulin family suggests that part of the fine tuning of the functions of α-tubulin and β-tubulin in microtubules is the result of the variation in the expression of different α- and β-tubulin genes in the different tissues, but, because there are fewer *TUBG* genes, γ-tubulin has to perform housekeeping functions that are more conserved among the species. 

In eukaryotes, the α-, β-, and γ-tubulins work together in the formation of microtubules. Heterodimers of α- and β-tubulin assemble microtubules, and two main complexes containing γ-tubulin—the γ-tubulin small complex (γ-TuSC) and the γ-tubulin ring complex (γ-TuRC)—assist in microtubule nucleation [5,6,7]. In humans, γ-TuSC consists of two γ-tubulin molecules in combination with one γ-tubulin complex protein 2 (GCP2) and with one GCP3. γ-TuSCs, together with additional GCPs, form the larger complex γ-TuRC. γ-TuSC and γ-TuRC are found in cytoplasm and centrosomes and are also associated with cellular membranes [8,9,10,11].

Here, we review and discuss the possible functions of γ-tubulin as a cellular meshwork based on recent advances in the field.

## 2. γ-Tubulin Is an Essential Protein

**Figure 1:**
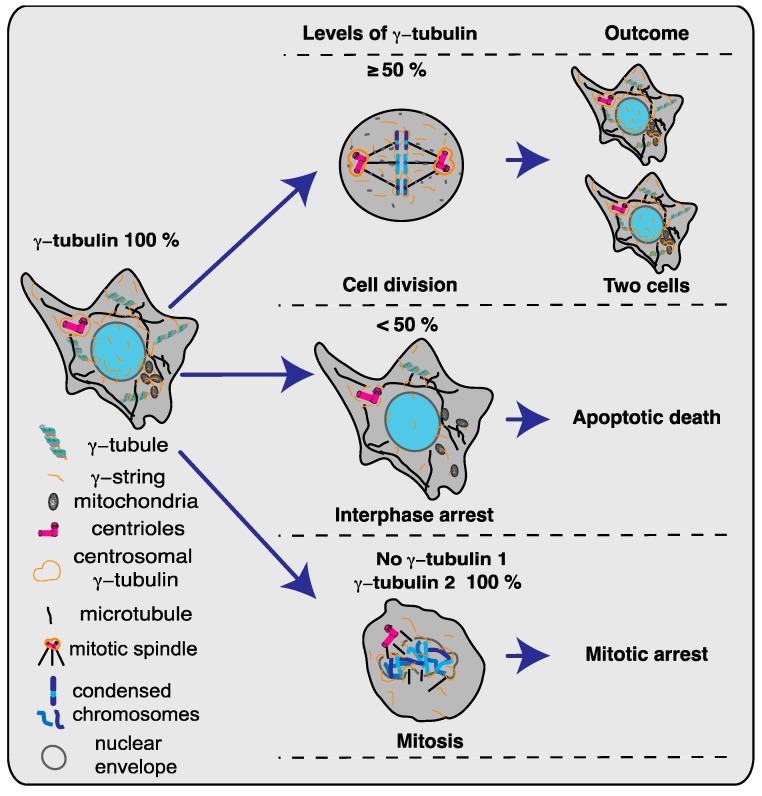
The levels and location of γ-tubulin protein that influences the proliferation capacity of mammalian cells. Schematic representation [22] showing how changes in the levels of the γ-tubulin protein (γ-tubulin 1 and γ-tubulin 2) in different cell compartments affect a proliferating cell. Cells with γ-tubulin levels of >50% divide normally, whereas those with levels of <50% arrest in interphase and ultimately die of apoptosis. Thereafter, selective depletion of γ-tubulin 1 protein reduces the pool of centrosome-associated protein, which leads to mitotic arrest due to impaired centrosome duplication and aberrant mitotic spindles.

To date, nothing is known about a species that lacks γ-tubulin, and about half of the studied species contain a single *TUBG* gene [3]. In mice, *TUBG1* is the predominantly expressed gene, although *TUBG2* is expressed up to the blastocyst stage during embryonic development and in the brain [8]. The *TUBG2*-knockout mice are viable and fertile, but exhibit some defects, including abnormalities in their circadian rhythm and in painful stimulation [8]. In contrast, the *TUBG1*-knockout mice survive to the morula/blastocyst stage. Cells from cultured *TUBG^−/−^* embryos show cell division arrest with unaligned and abnormally condensed chromosomes [8]. Additionally, in such embryonic cells, the microtubules are stable and the mitotic spindles are deformed and abnormal, carrying condensed and unaligned chromosomes, and the cells contain only one pericentrin (a centrosomal marker) rich center. These characteristics suggest that they have defects in the microtubules dynamics and centrosome duplication. Interestingly, *TUBG1^−/−^* embryos contain parental γ-tubulin 1 that is progressively diluted during the division of the fertilized egg. These embryos also express γ-tubulin 2, but the protein is not present in the centrosomes [8], which may result in disorganized spindles and the subsequent impairment of both nuclear division and centrosome replication (Figure 1). Inasmuch as similar phenotypes have been reported in other systems, the *TUBG* genes are considered essential [8,12,13,14,15,16,17,18,19,20,21].

Although γ-tubulin was regarded as a low-abundant protein when first discovered [17], today it is known that γ-tubulin is ubiquitously expressed in mammalian cells and appears in abundance in all cellular compartments [8,9,11,14,23,24,25,26,27]. In human and mouse cell lines, targeting the *TUBG1* and *TUBG2* with a single-guide (sg) or with short interference (si) RNA techniques induces a 50% drop in the levels of γ-tubulin protein, which leads to apoptotic death (Figure 1) [13,14,24,28]. The live imaging of *TUBG* sgRNA-expressing cells has demonstrated that when the levels of γ-tubulin decrease, cells divide normally for several days but subsequently they arrest in the interphase and die [14]. 

Based on previous work, it is surprising that the concomitant knockdown of both *TUBG* genes arrest cells in interphase and not in mitosis [8,15,16,17,19,21]. A plausible explanation for this effect is that the level of unconsumed parental γ-tubulin present in *TUBG* sgRNA-expressing cells is sufficient to aid execution of both interphase and mitosis, but, because each cell division halves the amount of γ-tubulin, the resulting γ-tubulin levels in the offspring cells cannot suffice to support interphase and thus the cells die in interphase. In contrast, in *TUBG1^−/−^* embryos, the amounts of parental γ-tubulin 1 and the expressed γ-tubulin 2 are enough to permit interphase progression, but, as the levels of parental γ-tubulin 1 drop, there is a concomitant decrease in the centrosome-associated pool of γ-tubulin, which results in mitotic failure [8]. Consequently, cell division in *TUBG*-knockdown cells will continue as long as the inherited γ-tubulin pool is sufficient to allow the execution of the following interphase [14], whereas cell division in *TUBG1^−/−^* embryos will proceed as long as the inherited centrosome-associated γ-tubulin pool is adequate for the execution of the subsequent mitosis (Figure 1) [8]. 

## 3. Is γ-Tubulin a Sticky Protein or a Meshwork?

Despite considerable progress in understanding the functions and location of γ-tubulin, the views regarding their functions have broadened. Initial genetic and biochemical experiments suggested that cellular γ-tubulin is either soluble or is associated with the following cellular structures: microtubule-organizing centers (centrosomes in animal cells and spindle pole bodies in fungi), the mitotic spindle, the kinetochore, midbodies, and microtubule arrays [29,30,31,32,33,34,35]. Different studies have found that part of the soluble γ-tubulin pool is in a complex with chaperonin containing TCP-1 (CCT), which is involved in the correct folding of tubulins [36,37]; part of it is in a complex with pericentrin, a cytosolic and pericentriolar matrix (PCM) protein [38]; and the rest of γ-tubulin is organized in either γ-TuSCs or γ-TuRCs [5,6,7]. Notably, γ-tubulin is detected in all biochemically divided fractions of cells and has, therefore, been considered to simply represent the contamination caused by the sticky nature of the protein. 

A number of studies in recent years have broadened this view of γ-tubulin [9,10,11,14,23,24,25,26,27,28,39,40,41,42,43]. In some cell lines and primary cells, a pool of γ-tubulin in the nuclear compartment [27,44,45] has been detected and found to be associated with Rad51, C53, GCP2, GCP3, E2F1, and lamin B1 [23,24,25,26,27,28]. Moreover, γ-tubulin affects the proper positioning and biogenesis of the Golgi apparatus, and it is also associated with endosomes and the endoplasmic reticulum [9,11,14]. Together, the reported data demonstrate that γ-tubulin is associated with all of the following: the nucleus, the Golgi, the endoplasmic reticulum, the mitochondria, the centrosomes, and the cytoplasm [9,10,11,14,25,27,44,45,46]. 

Within the PCM, γ-tubulin–pericentrin complexes form strings [38]. γ-Tubulin produced by bacteria assembles in vitro a meshwork of threads called γ-strings that support the formation of lamin B3 protofilaments [28]. Additionally, nuclear assembly experiments using extracts of *Xenopus laevis* eggs and mammalian cell lines have demonstrated that a nuclear boundary of γ-strings at the transition between cytosolic and chromatin-associated γ-tubulin serves as a supporting scaffold for the formation of the lamina and for the recruitment of the nuclear membrane [28]. An in vivo analysis of zebrafish embryos and in vitro experiments have shown that part of the cellular γ-tubulin pool is folded into γ-strings by the chaperonin CCT [39]. Experiments on the plant *Arabidopsis thaliana* have confirmed and further clarified the structure of γ-strings [43]. γ-Strings are 4 to 6 nm in diameter [14,28,39,43] and, in animal cells, span from the cytosolic compartment through the membranes and into the chromatin [14,28]. 

**Figure 2:**
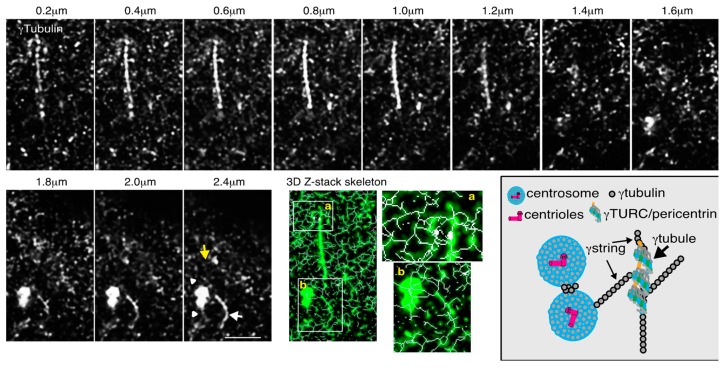
γ-Strings, γ-tubules, and centrosomes are interlinked to form a cellular meshwork. To determine whether γ-tubules are interconnected with centrosomes and γ-strings, and to capture the faintest signals emitted by thin γ-strings (Ø 4–6 nm), an Airyscan super-resolution microscopy was performed to reveal the details in the γ-tubulin meshwork. The U2OS cells were fixed, and endogenous γ-tubulin was immunostained with an anti-γ-tubulin antibody [48]. The sequential Airyscan super-resolution images were collected at 0.2-μm intervals. A 3D thinning algorithm was applied to the Z-stack images to find the centerlines (skeleton) of structures in the input images [49]. The projection of the obtained Z-stack-tagged skeleton is shown (3D Z-stack skeleton). The 3D Z-stack skeleton images illustrate γ-strings that emanated from both γ-tubules and centrosomes interlinking the meshwork [13]. White boxes indicate the areas magnified in the inset. The final figure is a schematic representation of the interlinking that γ-strings establish between γ-tubules and centrosomes. The white arrowheads, and white arrow, and the yellow arrow point out centrosomes, γ-tubules, and γ-strings, respectively. The illustrated images are representative of at least six experiments. Scale bar: 1 μm.

The γ-tubules represent another cellular structure that consists of γ-tubulin (Figure 2). γ-Tubules were first described as filaments formed upon the overexpression of γ-tubulin in monkey kidney Cos cells [47]. A recent characterization of γ-tubules in mammalian cells demonstrated that γ-tubulin assembles γ-tubules, which are cytosolic fibers containing γTuRCs and pericentrin [13]. In addition to γ-tubules and γ-strings, other γ-tubulin-rich structures are the centrosomes. In summary, the mentioned observations imply that γ-tubulin in mammalian cells is associated with all cellular compartments and, due to its self-polymerizing features, can be further organized in PCM, γ-strings, and γ-tubules. The cited findings also suggest that the sticky nature of γ-tubulin is the result of the self-polymerizing ability of the protein in combination with its presence in all cellular compartments.

### The γ-Tubulin Meshwork

Within an animal cell, the self-polymerizing ability of γ-tubulin results in the formation of γ-strings, and γ-tubules, and the γ-strings associated with the centrosome, which suggests that these components are interlinked to form a cellular meshwork in both the cytosol (including all cellular organelles) and the nuclear compartment. 

Z-stack images captured by Airyscan super-resolution microscopy, a technique known to improve the signal-to-noise detection of the faintest signals emitted by thin γ-strings (Ø 4–6 nm), were analyzed and, thereafter, 3D-skeleton images were generated by applying a 3D thinning algorithm to locate the centerlines (skeleton) of the structures in the input images [49]. This approach demonstrated that links are established between γ-strings, γ-tubules, and centrosomes (Figure 2). Considering that interlinking between filaments is the key to the formation of a skeleton, we propose that cellular γ-tubulin forms a meshwork. 

## 4. The Functions and Dynamics of the γ-Tubulin Meshwork

It is known that the functions of cytoskeletal and nucleoskeletal networks include providing form and mechanical support for a cell, organizing the genome, and assisting in signal transduction, cell movement, cellular transport, and cell–cell interactions [50]. However, how does the γ-tubulin meshwork contribute to cellular homeostasis? 

### 4.1. Providing Form and Mechanical Support, and Assisting in the Movement and Positioning of Organelles

It is plausible that centrosomes, γ-strings, and γ-tubules provide cells with mechanical support in various ways. In dividing cells, two centrioles are embedded in the PCM to form a centrosome. Interfering with γ-tubulin affects the centrosome duplication, which suggests that, in the PCM, γ-tubulin gives the parental centrioles with the mechanical support required for the formation of offspring centrioles [15,21,51]. Moreover, the PCM constitutes an important site that regulates the nucleation and the dynamics of microtubules and actin filaments during mitosis (i.e., a bipolar mitotic spindle ensures the strict segregation of sister chromatids between daughter cells) and also during interphase [30,52,53]. Furthermore, a boundary of γ-strings at the transition between the cytoplasm and the chromatin supports the formation of the lamina, and the recruitment of nuclear membranes, assuring the formation of a nuclear envelope around the chromatin [28]. In mitochondria and the Golgi apparatus, γ-strings produce a structuring scaffold that aids to the shaping and positioning of these organelles [11,14]. In addition, it is possible that membrane-associated γ-strings provide nucleating sites for microtubules, which would facilitate the movement and positioning of organelles in the cytoplasm. This notion is supported by a study showing that, in yeast cells, the Leu387Pro mutation in γ-tubulin influences nuclear positioning [54].

**Figure 3:**
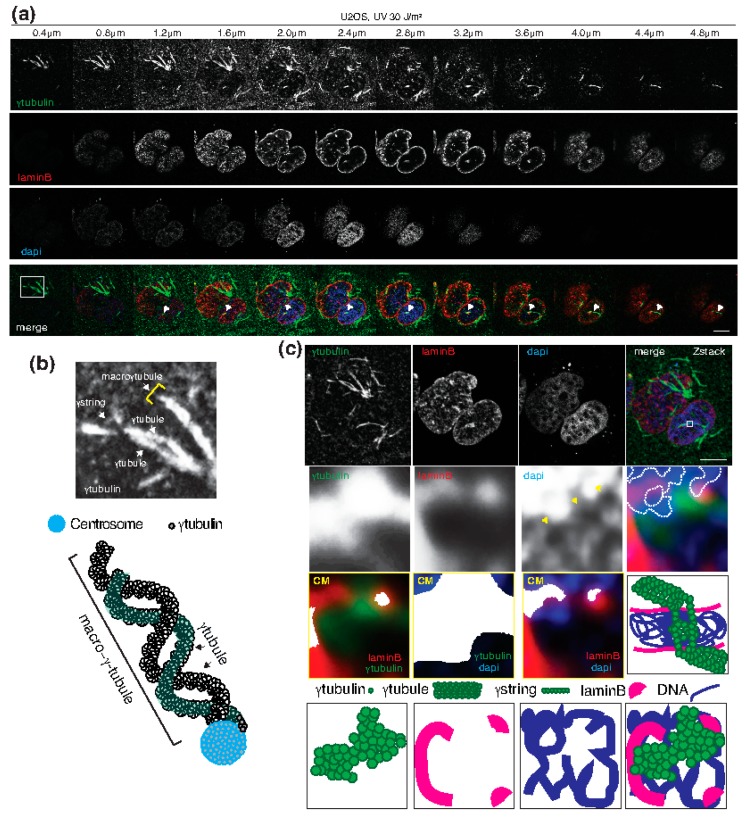
Connecting both sides of the cytoplasm with the nuclear compartment. (**a**–**c**) U2OS cells were treated with 30 J/m^2^ UV light and incubated for 5 minutes before fixation. Endogenous γ-tubulin and lamin B were detected by confocal immunofluorescence microscopy (Zeiss LSM 700 Axio Observer microscope with a Plan-Apochromat 63× NA 1.40 oil immersion objective) using an anti-γ-tubulin and an anti-lamin B antibody. Nuclei were detected with 4′,6-diamidino-2-phenylindole, dihydrochloride (DAPI) [48]. (**a**) Sequential fluorescence Z-stack images showing the γ-tubulin meshwork, the lamina (laminB), and chromatin. White arrowheads follow two γ-tubules that meet in the nuclear compartment. The images were collected at 0.4 μm intervals. The white box indicates the area magnified in the micrograph in panel (**b**). (**b**) The magnified area (micrograph) and a cartoon depicting two γ-tubules that produce a macro-γ-tubule by nucleating on a centrosome. The γ-tubulin molecules form strings (γ-strings) and γ-tubules. (**c**) Z-stack showing average intensity projection of the collected images in panel (**a**). The white box indicates the area magnified in the inset. Dense chromatin domains in the inset are designated with a dashed borderline and yellow arrowheads show less dense chromatin domains. Yellow boxes represent colocalized pixel maps (CM) of the indicated channels of the areas magnified in the inset. White areas in the maps denote pixels colocalized between channels [13]. Black boxes are schematic representations of the structures shown in the fluorescence images. The images in (**a**–**c**) are representative of at least six experiments. Scale bars: 10 μm.

Considering γ-tubules, these structures are approximately 25 nm in diameter and are most abundant in non-dividing cells, in which higher protein levels of the γ-tubulin protein are found in the cytoplasm [25,26,27,45,55]. Thus, a cell may control the cytosolic location of γ-tubulin through the formation of cytosolic γ-tubules. However, the centrosome is not a prerequisite for γ-tubule formation [13], although γ-tubules are found emanating from centrosomes at sites where the nuclear envelope is folded, and two γ-tubules are interlaced, to produce a macro-γ-tubule (Figure 3a,b). The creation of macro-γ-tubules may influence the shape of the nuclear envelope, a deduction that supports the role of γ-tubules as elements that alter the shape of the nuclear compartment.

### 4.2. Assisting in Signal Transduction, and Organizing the Genome 

There is substantial evidence that the γ-tubulin meshwork regulates the G1-to-S transition [24,25,56,57,58,59,60,61,62,63,64], mitotic progression [65], and cytokinesis [66], effects that are achieved by harboring of various proteins involved in the signal transduction pathways that coordinate cell cycle progression. Additionally, the PCM acts as a signal hub that brings together various checkpoint proteins [24,25,56,57,58,59,60,61,62,63,64,65,66,67]. A more detailed review of the involvement of γ-tubulin in signal transduction has recently been published [22]. 

The spatial organization of the genome regulates gene expression [68], and both genome organization and gene expression may be controlled by the γ-tubulin meshwork. In this context, γ-tubulin binds to the DNA on the same DNA binding motif as E2 promoter-binding factor (E2F), which results in that the retinoblastoma (RB1) and γ-tubulin proteins moderate each other´s expression by binding to their respective gene promoters. Thus, the reduction of the levels of the γ-tubulin protein results in an up-regulation of the expression of the RB1 protein [24,25]. This suggests that RB1 and γ-tubulin may complement each other in the regulation of gene expression, where the lack of activity/expression of one protein may be compensated for by an increase in activity/expression of the other protein. Indeed, the RB1/γ-tubulin regulatory network controls E2Fs [25], which transcriptional activities are necessary to induce the expression of the target genes that are essential for centrosome duplication and DNA replication [69]. Interestingly, in addition to interacting with E2Fs, RB1 modifies the structure and organization of chromatin by recruiting remodeling factors, including histone deacetylases, members of the chromatin remodeling complex SWI/SNF, and DNA methyltransferase [70,71,72], which indicates the potential involvement of γ-tubulin in the recruitment of DNA-remodeling factors. 

Considering that γ-strings are present in the double membranes of the nuclear envelope and of the mitochondria [14,28], as well as in the chromatin, and that lamin B is capable of nucleating on γ-strings, it is tempting to speculate on the possible mechanisms by which the γ-tubulin meshwork can assist in signal transduction and genome organization. A confocal microscopy analysis of fixed human U2OS osteosarcoma cells revealed that two γ-tubules are connected through the nuclear compartment and thereby link both sides of the cytosol (Figure 3a,c). A closer analysis of Z-stack images captured with a confocal microscope showed that, at the γ-tubule connecting site inside the nuclear compartment, the chromatin is less dense and appears to have holes (Figure 3c). Chromatin-dense areas or domains can also be seen in the vicinity of γ-tubulin (Figure 3c). These findings agree with the possibility that cytosolic γ-tubules serve as structural docking sites for nuclear γ-tubulin, resulting in the remodeling of the genome and in the formation of docking sites that might be used for the recruitment of chromatin-remodeling factors.

### 4.3. The Dynamics of the γ-Tubulin Meshwork

The γ-tubulin meshwork changes in a cell-cycle-dependent manner. In the G1 phase, most of the γ-tubulin pool is in the cytosol, and part of this cytosolic pool creates γ-tubules (Figure 2) [13]. The formation of γ-tubules is determined by a GTP-dependent balance between γ-tubules and γ-tubulin [13]. However, at the G1–S transition, the number of cytosolic γ-tubules is reduced, and there is a subsequent accumulation of γ-tubulin in the nuclear compartment and the PCM [13,21,25]. In the S phase, the inherited centrosome and genome duplicate synchronously. At the onset of mitosis, the two centrosomes ensure the assembly of a bipolar mitotic spindle and the strict segregation of sister chromatids between daughter cells. In addition, a boundary of γ-strings ensures the formation of the nuclear envelope around chromatin [28]. 

Studies of murine NIH3T3 embryonic fibroblasts and human U2OS osteosarcoma cells have identified a bipartite nuclear localization signal (NLS) on the C terminus of γ-tubulin [55]. Notably, SadB kinases (e.g., mSADB and hSAD1/BRSK1) mediate the phosphorylation of γ-tubulin on Ser^385^, a residue that is near the NLS and also occurs in the starting region of a motif, which is commonly found in DNA-binding proteins and is called a helix-loop-helix [22]. The phosphorylation levels of γ-tubulin on Ser^385^ induce a conformational change to unmask the NLS, which leads to the nuclear accumulation of γ-tubulin [21,23,24,25,26,27,55]. The DNA-binding ability of the C terminus of γ-tubulin is also important for the association of the protein with mitochondrial DNA [14,25]. Moreover, early in the S-phase, SadB kinases mediate the phosphorylation of γ-tubulin on Ser^131^ [21,51,55]. The phosphorylation levels of γ-tubulin on Ser^131^ regulate the recruitment of γ-tubulin at the nascent centriole and facilitate the accessibility of SadB to Ser^385^ [55]. Another biochemical modification that affects the γ-tubulin meshwork is the BRCA1-dependent ubiquitination of γ-tubulin, which regulates the centrosome number [73].

In contrast to γ-strings, which are static structures, γ-tubules vary in length and nucleate on cytosolic γ-tubulin foci together with pericentrin, GCP2, GCP3, GCP5, and GCP6. γ-Tubules are affected by the following: the methodology used for fixation of cells; treatment with cold, colcemid, taxol, citral dimethyl acetal (CDA), and dimethyl fumarate (DMF); and the expression of γ-tubulin mutants that affect the GTPase domain of γ-tubulin [13,48]. Intriguingly, both the citral analogue CDA and the approved drug DMF bind to the GTPase domain of γ-tubulin, which affects the length of γ-tubules, the nuclear activity of γ-tubulin, and the respiratory capacity of mitochondria [14,56,74]. Accordingly, in human U2OS osteosarcoma cells, mutations in the GTP/magnesium-interacting residue Cys^13^ of γ-tubulin are cytotoxic [13,14,75,76]. These observations strongly suggest that the GTPase domain of γ-tubulin is essential for the dynamics of the γ-tubulin meshwork. The where and when of the self-polymerizing ability of γ-tubulin is most likely regulated by GTP acting together with phosphorylation-dependent changes in the conformation of γ-tubulin.

## 5. Conclusions and Future Perspectives

Recent advances support the notion that γ-tubulin is the main component of a cellular meshwork that plays a major role in cell survival. The aim of the present review is to unite recent findings in this field with previous knowledge of the protein to summarize the known functions of γ-tubulin in cellular homeostasis, and also to speculate on the possible roles and organization of the meshwork. Nonetheless, further insights are still needed to elucidate the mechanical signals affecting the spatial organization of the γ-tubulin meshwork and the molecules controlling the dynamics of this meshwork. Importantly, γ-tubulin is related to several human diseases. Levels of γ-tubulin are increased in various tumors, and inhibition of the GTPase activity of γ-tubulin in RB1-deficient tumor cells impedes tumor growth [24,25,56,77,78,79,80,81,82]. Mutations in the *TUBG* genes have also been found to cause brain malformations [54,83]. Finally, DMF is a γ-tubulin inhibitor used to treat multiple sclerosis and psoriasis, both of which are autoimmune diseases [56,74,84,85]. Consequently, knowledge concerning the γ-tubulin meshwork is required to highlight the potential roles of γ-tubulin in diseases and development, and to aid in the discovery of novel therapeutic regimens that target the activity of γ-tubulin.

## Abbreviations

γ-TuSC	γ-Tubulin small complex
γ-TuRC	γ-Tubulin ring complex
siRNA	Short interference RNA
sgRNA	Single-guide RNA
CCT	Chaperonin-containing TCP-1
DMF	Dimethyl fumarate
CDA	Citral dimethyl acetal
NLS	Nuclear localization signal
PCM	Pericentriolar matrix
GCP	γ-Tubulin complex protein
E2F	E2 promoter-binding factor
RB1	Retinoblastoma protein
